# Anxiolytic Drug FGIN-1-27 Ameliorates Autoimmunity by Metabolic Reprogramming of Pathogenic Th17 Cells

**DOI:** 10.1038/s41598-020-60610-5

**Published:** 2020-02-28

**Authors:** Anju Singh, Myagmarjav Dashnyam, Bryan Chim, Thelma M. Escobar, Andrés E. Dulcey, Xin Hu, Kelli M. Wilson, Prasanthi P. Koganti, Camille A. Spinner, Xin Xu, Ajit Jadhav, Noel Southall, Juan Marugan, Vimal Selvaraj, Vanja Lazarevic, Stefan A. Muljo, Marc Ferrer

**Affiliations:** 1 Division of Preclinical Innovation, National Center for Advancing Translational Sciences (NCATS), National Institutes of Health, Rockville, MD USA; 2 Laboratory of Immune System Biology, National Institute of Allergy and Infectious Diseases, NIH, Bethesda, MD USA; 3 Department of Animal Science, College of Agriculture and Life Sciences, Cornell University, Ithaca, New York USA; 4 Experimental Immunology Branch, National Cancer Institute, National Institutes of Health, Bethesda, MD USA

**Keywords:** Drug discovery, Immunology

## Abstract

Th17 cells are critical drivers of autoimmune diseases and immunopathology. There is an unmet need to develop therapies targeting pathogenic Th17 cells for the treatment of autoimmune disorders. Here, we report that anxiolytic FGIN-1-27 inhibits differentiation and pathogenicity of Th17 cells *in vitro* and *in vivo* using the experimental autoimmune encephalomyelitis (EAE) model of Th17 cell-driven pathology. Remarkably, we found that the effects of FGIN-1-27 were independent of translocator protein (TSPO), the reported target for this small molecule, and instead were driven by a metabolic switch in Th17 cells that led to the induction of the amino acid starvation response and altered cellular fatty acid composition. Our findings suggest that the small molecule FGIN-1-27 can be re-purposed to relieve autoimmunity by metabolic reprogramming of pathogenic Th17 cells.

## Introduction

In recent years, a subset of CD4^+^ T cells, namely Th17 cells, which are characterized by production of the signature cytokines IL-17 (IL-17A), IL-17F and IL-22 has been identified^1–3^. These Th17 cells play a critical role in promoting mucosal immunity and protection against fungal pathogens (e.g. *Candida albicans*) and extracellular bacteria (e.g. *Staphylococcus aureus*)^4,5^. However, dysregulated Th17 cells can drive inflammatory tissue pathology leading to autoimmune disorders such as inflammatory bowel disease, psoriasis, rheumatoid arthritis and multiple sclerosis. Multiple groups have reported that the cytokines IL-6 and TGF-β can initiate differentiation and lineage commitment of naïve CD4^+^ T cells to Th17 cells^2,6^. However, exposure to pro-inflammatory cytokine IL-23 plays a crucial role in inducing pathogenicity leading to development of pathogenic Th17 cells with a unique inflammatory signature^3,7,8^.

T cell activation and differentiation requires signals from the T cell receptor (TCR, signal 1), co-stimulatory molecules (signal 2) as well as signals from various cytokines^9,10^. Activated T cells undergo profound metabolic reprogramming to meet the increased energy demands of rapid growth and differentiation^11,12^. Recent evidence has linked distinct metabolic programs with differentiation and maintenance of different T cell subsets. For instance, Th17 cells are highly glycolytic and rely on large quantities of glucose to meet their energy needs whereas regulatory T cells (Tregs) primarily rely on oxidative phosphorylation for their survival, proliferation and maintenance^13,14^. Targeting distinct metabolic programs utilized by different T cell sub-populations offers a novel way to regulate the desired immune responses.

By analyzing publicly available data sets, we found that Th17 cells expressed higher levels of TSPO compared to other T cell subsets, namely Th1, Th2 and regulatory T cells^15^ (Fig. S1a). We hypothesized that we could potentially target TSPO in Th17 cells since several small molecule ligands of TSPO had already been reported but have not been explored in this context^16^. For example, FGIN-1-27 [N, N-di-n-hexyl 2-(4-fluorophenyl) indole-3-acetamide)], one such ligand was initially developed as a tracer for diagnostic imaging for neuroinflammation and neurodegeneration^16–18^. It has been reported that FGIN-1-27 can cross the blood brain barrier and produce anti-anxiety and anti-panic effects by stimulating steroidogenesis of neuroactive steroids such as allopregnanolone^16–18^.

We found that FGIN-1-27 is a potent repressor of IL-17 production and Th17 differentiation program. However, using Th17 cells from TSPO knockout mice (TSPO KO), we demonstrate that the effect of FGIN-1-27 on Th17 cell differentiation is independent of TSPO. We further show that FGIN-1-27 reprograms Th17 cells by inducing a metabolic switch characterized by the activation of the amino acid starvation response (AAR), significant alterations in the cellular lipidome and downregulation of glycolytic enzymes and glycolytic intermediates. Importantly, FGIN-1-27 reduced the pathogenicity of Th17 cells in a mouse model of multiple sclerosis, demonstrating that small molecule FGIN-1-27 could be used therapeutically to ameliorate autoimmunity by inducing a metabolic switch in Th17 cells.

## Results

### FGIN-1-27 inhibits Th17 cell differentiation

**Figure 1:**
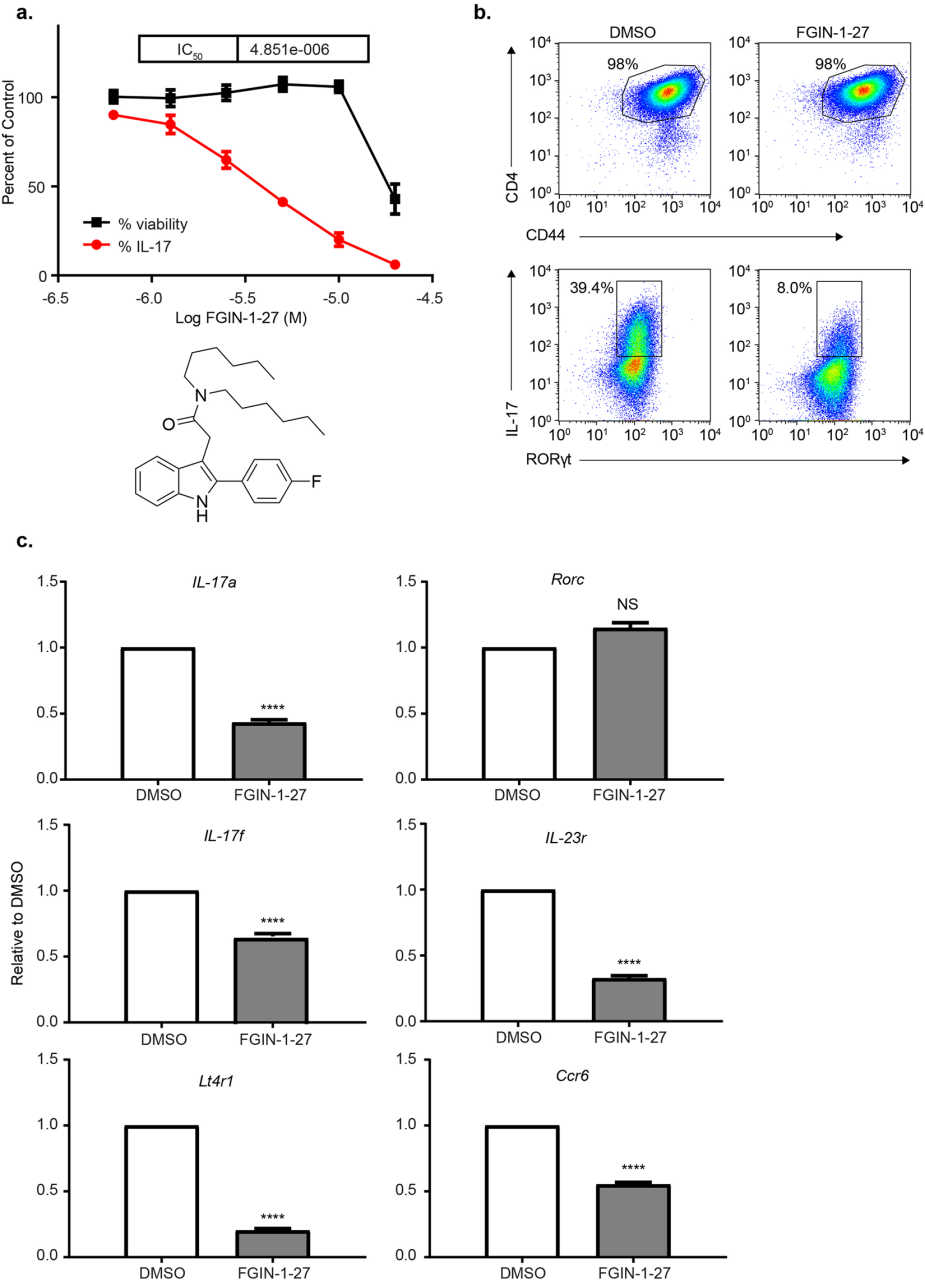
FGIN-1-27 Inhibits Th17 Differentiation. Naïve CD4 T cells from spleens and lymph nodes of C57BL/6 mice were differentiated under Th17 polarization conditions and were treated with DMSO (control) or FGIN-1-27. (**a**) Dose response plot showing effect of FGIN-1-27 on intracellular IL-17 and viability on day 4 post activation quantified by flow cytometry. Data is normalized against DMSO (100%). Bottom panel shows the structure of FGIN-1-27. (**b**) Dot plots showing DMSO or FGIN-1-27 treated cells stained for surface CD4, CD44, intracellular IL-17 and intranuclear RORγt. (**c**) Transcript levels for the indicated genes after 24 hours of treatment with DMSO or FGIN-1-27 quantified by qPCR. Data was normalized to a housekeeping gene, β-actin. Data are representative of at least 3 independent experiments and the error bars indicate standard deviation.

Because of differential TSPO expression in Th17 cells (Fig. S1a) and up-regulation of TSPO in neuroinflammation, we tested whether TSPO ligands, such as FGIN-1-27, can affect Th17 differentiation. In this assay, purified CD4^+^ T cells from mouse spleens and lymph nodes were cultured under Th17 polarizing conditions. Compared to DMSO treated cultures, treatment with FGIN-1-27 inhibited generation of IL-17 producing CD4^+^ T cells in a dose-dependent manner and abolished 85% of IL-17 producing cells without compromising viability (Fig. 1a,b). The effect on the Th17 differentiation program was not due to a defect in T cell activation because CD4^+^ T cells from FGIN-1-27 treated cultures up-regulated CD69 and CD44 to similar levels as DMSO treated T cells (Fig. S1b). However, proximal TCR signaling was affected as FGIN-1-27 treated cells did not phosphorylate ZAP70 on anti-CD3/CD28 stimulation compared to DMSO treated cells (Fig. S1c). Activated T cells from compound treated cultures had no defect in cell size and proliferated to the same extent as DMSO treated cells (Fig. S1d,e). FGIN-1-27 treatment had no effect on protein or transcript levels of RORγt, the Th17 lineage-determining transcription factor. However, FGIN-1-27 significantly downregulated the expression of RORγt target genes (all *p* < *0.0001*), notably *Il17a*, *Il17f*, *Il23r*, *Ltb4r1*,*Ccr6* (Fig. 1b,c) and *Stat3*. Notably, FGIN-treated Th17 cells did not show a defect in phosphorylation of STAT3 following re-stimulation with IL-6 (Fig. S1f).

We also tested whether FGIN-1-27 regulated IL-17 production in fully differentiated Th17 cells. To this end, polarized Th17 cells were treated with FGIN-1-27, and on re-stimulation fewer IL-17 producing cells were detected showing that FGIN-1-27 treatment could be effective in a therapeutic setting as well (Fig. S1h). Thus, FGIN-1-27 inhibits Th17 cell differentiation *in vitro* without compromising T cell activation or survival.

### FGIN-1-27 protects mice against EAE

**Figure 2:**
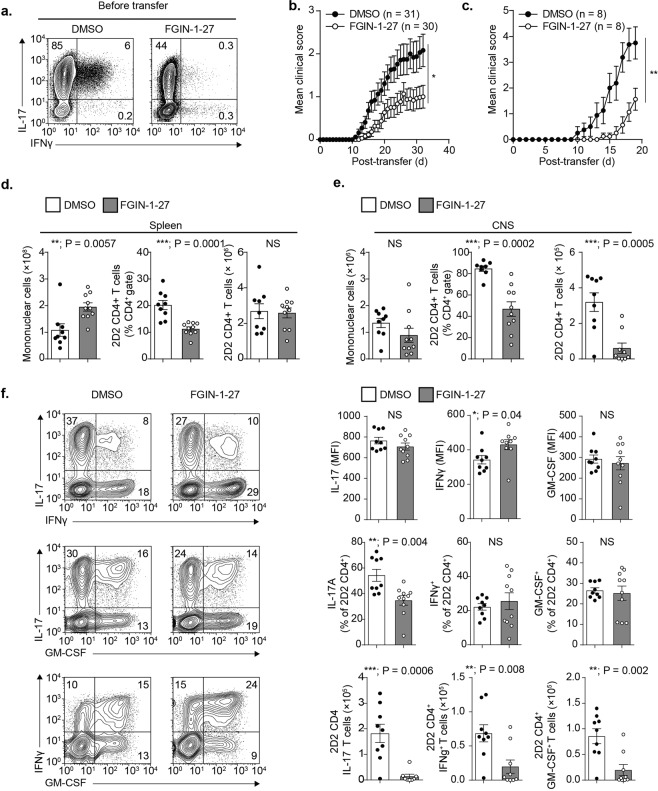
FGIN-1-27 protects mice against EAE. (**a**) Immunophenotyping of 2D2 TCR transgenic CD4^+^ T cells for intracellular IL-17 and IFNγ before adoptive transfer. Cultures were treated with DMSO or FGIN-1-27 during Th17 polarization process. (**b**) Mean clinical scores of mice following the adoptive transfer of 5 × 10^6^ 2D2 Th17 cells treated with DMSO or 5 × 10^6^ 2D2 Th17 cells treated with FGIN-1-27. Data are pooled from 31 recipient mice that received DMSO treated cells and 30 mice that received FGIN-1-27 treated cells (*p* < *0.02*). The error bars represent SEM. (**c**) Mean clinical scores of T cell-deficient (*Tcrb*^*−/−*^) mice following adoptive transfer of 5 × 10^6^ 2D2 Th17 cells treated with DMSO or 5 × 10^6^ 2D2 Th17 cells treated with FGIN-1-27. Data are pooled from 8 recipient mice for each group that received either DMSO or FGIN-1-27 treated cells (*p* = *0.0067*). The error bars represent SEM. (**d**) Enumeration of total mononuclear or 2D2 CD4^+^ T cells (CD4^+^V_β_11^+^) in spleens of mice receiving either DMSO or FGIN-1-27 treated 2D2 T cells. Cells were analyzed at the peak of EAE disease (day 15 post-transfer) by flow cytometry. The error bars represent SD. (**e**) Enumeration of total CNS mononuclear or CNS-infiltrating 2D2 CD4^+^ T cells (CD4^+^V_β_11^+^) in mice receiving either DMSO or FGIN-1-27 treated 2D2 T cells. Cells were analyzed at the peak of EAE disease (day 15 post-transfer) by flow cytometry. The errors bars represent SD. (**f**) Intracellular cytokine staining of 2D2 CD4^+^ T cells (day 15 post-transfer) for IL-17, IFNγ and GM-CSF from the CNS of mice that received either DMSO or FGIN-1-27 treated cells. The error bars represent SD.

We interrogated whether FGIN-1-27 can influence Th17 dependent pathology *in vivo* by using a mouse model of passive EAE in which Th17 cells are responsible for driving immuno-pathogenesis. To this end, we sorted naïve CD4^+^ T cells (CD4^+^V_β_11^+^CD62L^hi^) from spleen and lymph nodes of “2D2” T cell receptor (TCR) transgenic mice that specifically recognize myelin oligodendrocyte glycoprotein (MOG) peptide and activated them under Th17 polarizing conditions. The cultures were treated with DMSO or FGIN-1-27 during the Th17 differentiation process and cells were reactivated on day 5 for an additional 48 hours and equal numbers of 2D2 Th17 polarized cells treated either with DMSO or FGIN-1-27 were adoptively transferred to recipient mice. We characterized the cells pre-transfer and the FGIN-1-27 treated 2D2 T cells had a reduced percentage of IL-17 producing cells as well as cells that could make both IFNγ and IL-17 on re-stimulation (Fig. 2a).

Following the adoptive transfer of equal number of 2D2 Th17 cells, recipient mice that received FGIN-1-27 treated cells exhibited much milder disease symptoms, as shown by the mean clinical scores (Fig. 2b). Transfer of FGIN-1-27 treated 2D2 transgenic Th17 cells to T cell deficient (*Tcrb*^−/−^) recipient mice also led to a delayed onset and reduced severity of EAE, ruling out the possibility of compromised fitness and/or the survival of FGIN-1-27 treated transferred 2D2 Th17 cells within immunocompetent hosts, where the transferred cells must compete for homeostatic survival signals with endogenous T cells (Fig. 2c). Interestingly, we recovered fewer 2D2 CD4^+^ T cells from the CNS of mice and higher number of 2D2 CD4^+^ T cells from the spleens of mice that received FGIN-1-27 treated cells, suggesting that FGIN-1-27 treatment impairs the trafficking and/or CNS parenchymal invasion. (Fig. 2d,e). To better understand the effects of FGIN on the effector function of pathogenic Th17 cells, we evaluated the ability of the few FGIN-1-27 treated, CNS-infiltrating Th17 cells to produce cytokines by intracellular cytokine staining. In addition to recovering fewer 2D2 CD4^+^ T cells, we detected a significant reduction in the percentage and absolute numbers of IL-17 producing 2D2 CD4^+^ T cells isolated from the CNS of mice that received FGIN-1-27 treated cells (Fig. 2f). While IL-17 responses were selectively inhibited by FGIN-1-27 treatment, IFNγ and GM-CSF production were intact (Fig. 2f). We also tested FGIN-1-27 in an active model of EAE where EAE was induced by immunization with the myelin oligodendrocyte glycoprotein (MOG) peptide 35-55. Mice were injected with vehicle or FGIN-1-27 for 7 days and classical EAE symptoms were scored daily according to standard criteria (Fig. S1i). Treatment with FGIN-1-27 did not affect onset or severity of the symptoms in an active EAE model leading us to speculate that multiple cell types besides Th17 might be affected in an active model and FGIN-1-27 might be specifically affecting Th17 differentiation. Collectively, these data demonstrate that FGIN-1-27 can be effectively used to selectively target Th17 differentiation both *in vitro* and in an *in vivo* autoimmune disease setting where FGIN-1-27 abrogated the pathogenic potential of Th17 cells and limited CNS pathology.

### Effect of FGIN-1-27 on Th17 Differentiation is Independent of TSPO

**Figure 3:**
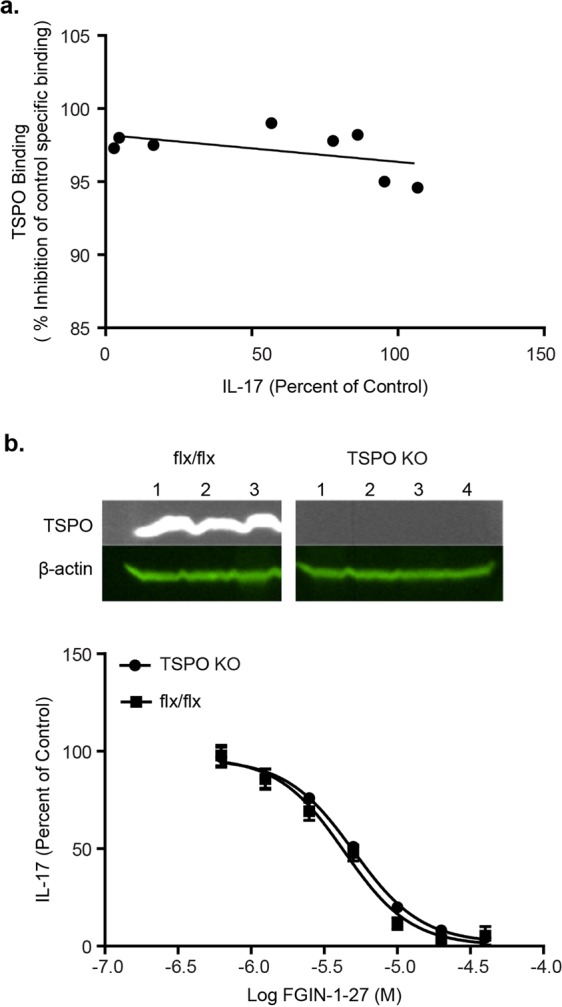
Effect of FGIN-1-27 on Th17 Differentiation is Independent of TSPO. (**a**) Plot showing correlation between binding of FGIN-1-27 to TSPO and effect on IL-17 production. Binding of FGIN-1-27 to TSPO was measured using a biochemical radiolabeled displacement assay and radiolabeled PK-11195 was used as a control ligand. (**b**) Immunoblot to detect TSPO from spleens of either control or TSPO knockout mice (upper panel) and dose response plot for FGIN-1-27 showing effect on Th17 differentiation using purified CD4 T cells from spleens of either control or TSPO knockout mice. IL-17 production was normalized using DMSO treatment from control cells as 100%. Data are representative of 3 independent experiments and the error bars represent SD.

We explored the mechanism of action for FGIN-1-27 and whether the effect of FGIN-1-27 on Th17 differentiation was driven by TSPO, the reported target of this compound. We used two approaches: first, we investigated the correlation between FGIN-1-27 induced IL-17 down-regulation and binding to TSPO. For the binding studies, we used a biochemical radio ligand displacement assay with the well-characterized TSPO ligand PK-11195 as a tracer. FGIN-1-27 displaced PK-11195 at all concentrations tested (0.3–40 µM), and the concentration response curve showed more than 90% displacement at the lowest validated testing concentration (0.3 µM) showing that FGIN-1-27 binds TSPO with high affinity (Fig. S1j). However, binding of FGIN-1-27 to TSPO did not correlate with its effect on IL-17 production (Fig. 3a). Secondly, we purified CD4^+^ T cells from spleen and lymph nodes of mice that lacked global expression of TSPO (TSPO KO mice)^19^. We added FGIN-1-27 to the cultures of CD4^+^ T cells from controls or TSPO KO mice that were induced towards the Th17 differentiation program. FGIN-1-27 downregulated Th17 differentiation in T cells from TSPO KO mice similarly to T cells expressing TSPO, demonstrating that the effect of FGIN-1-27 on Th17 differentiation is independent of TSPO (Fig. 3b).

### Th17 cells undergo metabolic reprogramming upon FGIN-1-27 treatment

**Figure 4:**
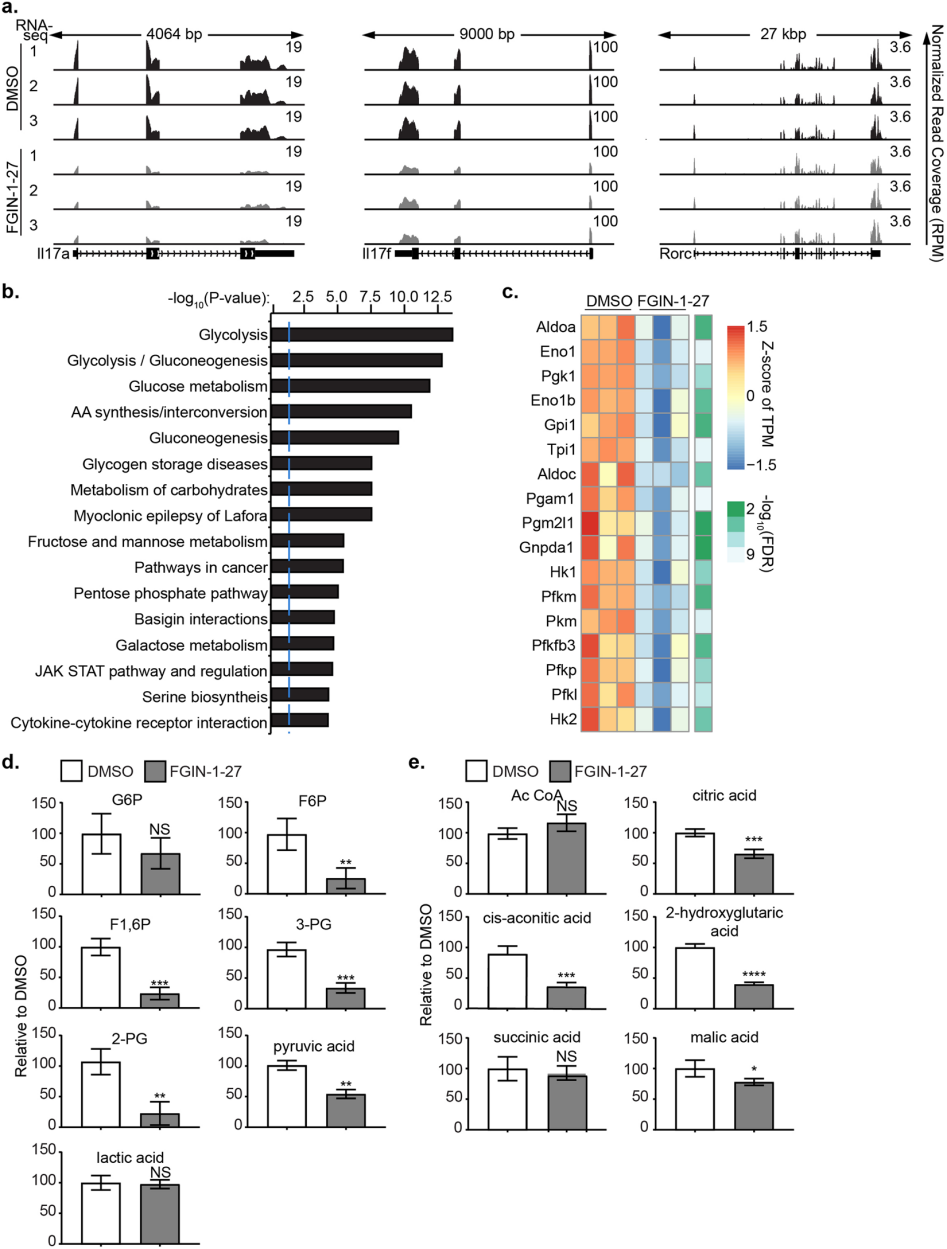
Metabolic Reprogramming of Th17 cells on FGIN-1-27 Treatment. CD4 T cells were differentiated under Th17 polarization conditions and were either treated with DMSO (control) or FGIN-1-27. The error bars represent SD. (**a**) Genomic browser screenshots of IL-17a, IL-17f and Rorc loci depicting normalized RNA-seq read coverage (RPM) in Th17 cells treated with DMSO or FGIN-1-27 for 24 hours. (**b**) Bar plots showing negative log_10_ P-values of the 16 most enriched pathways from the INOH, KEGG and Reactome databases, as calculated by InnateDB using the 587 differentially expressed genes between DMSO and FGIN-1-27 RNA-seq as input. (**c**) Heat map showing differentially expressed genes encoding glycolytic enzymes in Th17 cells treated with DMSO or FGIN-1-27 for 24 hours (**d**) Glycolytic metabolites measured in Th17 cells treated with DMSO or FGIN-1-27 for 72 hours. (**e**) Metabolites in the TCA cycle were measured in Th17 cells treated with DMSO or FGIN-1-27 for 72 hours.

Results from FGIN-1-27 treatment of T cells from TSPO KO mice led us to explore for cellular mechanisms by which this drug prevented Th17 cell differentiation. We investigated the possible molecular mechanism(s) regulating impaired Th17 differentiation using whole-transcriptome RNA sequencing (RNA seq) of CD4^+^ T cells treated with FGIN-1-27 under Th17 polarizing conditions. Gene expression analysis showed that out of 587 differentially expressed genes, 332 were down-regulated and 256 were up-regulated on treatment with FGIN-1-27 compared to DMSO treated cells (FDR < 0.05). We confirmed down-regulation of IL-17 cytokines (IL-17a and IL-17f) in FGIN-1-27 treated cells as well as no effect on *Rorc* gene expression after treatment (Fig. 4a).

Pathway enrichment analysis showed that metabolic pathways, such as glycolysis, were preferentially enriched on FGIN-1-27 treatment (Fig. 4b). Genes encoding enzymes in the glycolytic pathway such as hexokinase, phosphofructokinase, and pyruvate kinase were significantly down-regulated in FGIN-1-27 treated CD4 T cells (Fig. 4c). Metabolic profiling in FGIN-1-27 treated CD4^+^ T cells showed that upper glycolytic intermediates, such as fructose 6 P (F6P), fructose 1,6 P (F1,6P), 3 phosphoglyceric acid (3-PG), 2 phosphoglyceric acid (2-PG), were substantially reduced compared to DMSO treated controls (Fig. 4d). Levels of late glycolytic intermediates were not changed, and FGIN-1-27 treated cells had lower levels of pyruvate, but unchanged levels of lactate, suggesting preferential conversion of pyruvate to lactate.

Further metabolomic analysis revealed that TCA cycle intermediates, such as citric acid, cis-aconitic acid, 2-hydroxyglutaric acid and malic acid, were additionally reduced on FGIN-1-27 treatment (Fig. 4e). However, levels of acetyl-coA in FGIN-1-27 treated Th17 cells were comparable to DMSO, primarily because of decreased oxidative phosphorylation (Fig. 4e). Thus, FGIN-1-27 treatment alters the metabolic profile of T cells by reducing upper glycolytic and TCA intermediates in Th17 cells.

### FGIN-1-27 induces amino acid starvation response (AAR)

**Figure 5:**
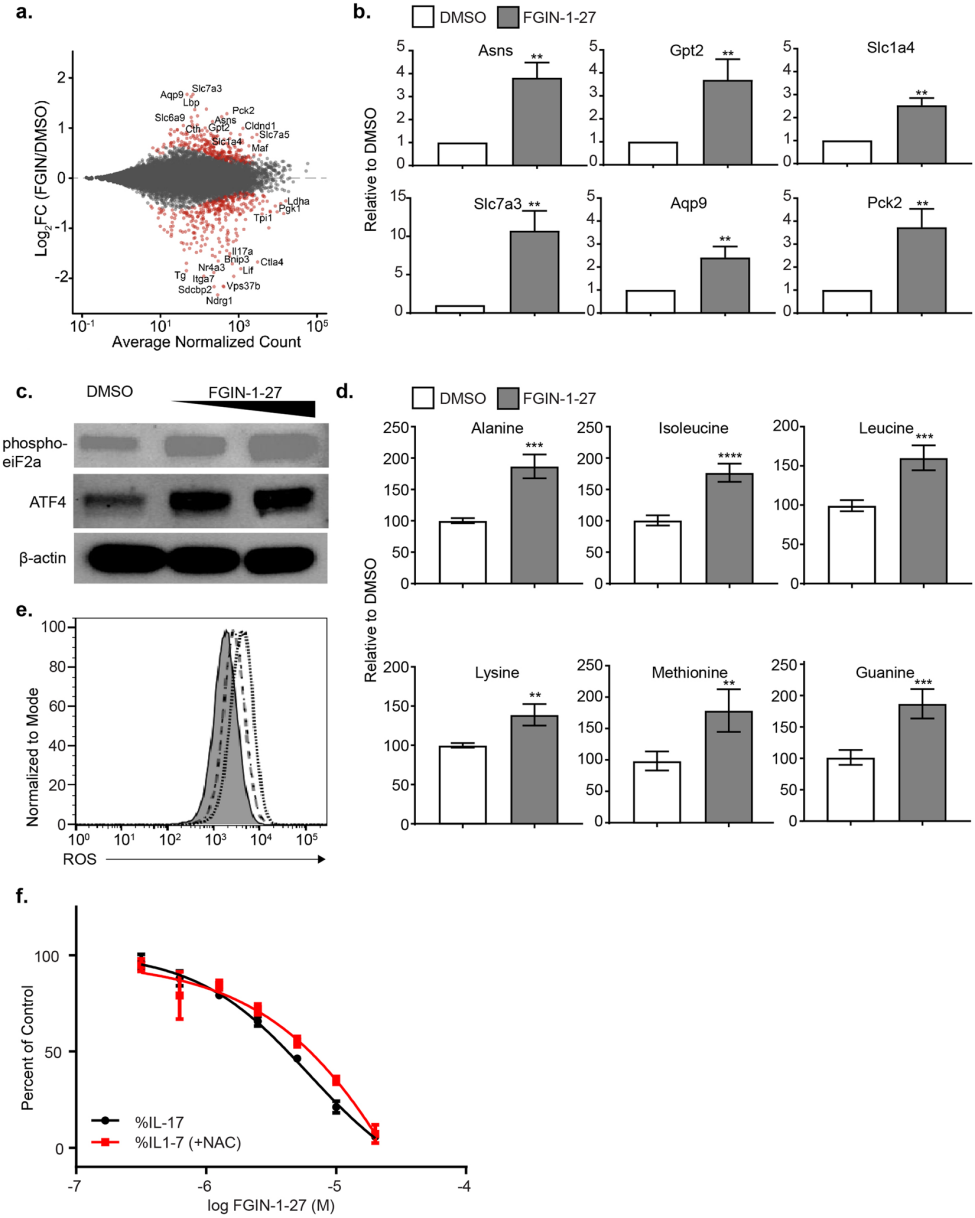
FGIN-1-27 induces amino acid starvation response (AAR). (**a**) MA-plot showing shrunken log_2_ fold-changes of detected genes between the DMSO and FGIN-1-27 RNA-seq samples, ranked by average normalized count. Red dots indicate differentially expressed genes at adjusted p-value <0.05. (**b**) Transcript levels for the indicated genes after 24 hours of treatment with DMSO or FGIN-1-27 quantified by qPCR. The error bars represent SD. (**c**) Western blot analysis for phosphorylated eIF2α and ATF4 24 hours after treatment with DMSO or FGIN-1-27 (5 and 10 µM). Data are representative of 3 independent experiments. (**d**) Amino acids in Th17 cells measured 72 hours after treatment with FGIN-1-27. The error bars represent SD. (**e**) ROS was measured using cell permeant reagent 2′,7′ –dichlorofluorescin diacetate (DCFDA) at 24 hours following treatment with FGIN-1-27. Filled grey histogram-DMSO, dotted line-FGIN-1-27 (10 µM) and dashed line indicates FGIN-1-27 (5 µM). (**f**) Dose response plot showing effect of FGIN-1-27 on intracellular IL-17 after pre-treatment with NAC (1 µM). Data are normalized using DMSO treated cells as 100% and the error bars represent SD.

In addition to glycolysis, transcriptomic analysis showed that amino acid synthesis/interconversion was the second major pathway affected by FGIN-1-27 treatment of T cells, and many of the differentially expressed transcripts induced upon FGIN-1-27 treatment were associated with either amino acid transport or biogenesis (Figs. 4b and 5a). RT-qPCR validation revealed that ATF4 target genes, asparagine synthetase (*Asns*) and glutamic pyruvate transaminase (*Gpt2*) that are involved in amino acid synthesis and anaplerotic reactions, were induced by FGIN-1-27 (Fig. 5a,b). Amino acid transporters, such as Slc1a4 (aka *Asct1*) and Slc7a3, as well as glycerol transporters, such as aquaporin 9 (Aqp9), were also up-regulated by FGIN-1-27. Cataplerotic enzyme, phosphoenopyruvate carboxylase 2 (*Pck2*) that catalyzes generation of PEP (a precursor for serine synthesis) from TCA metabolite oxaloacetate (OAA) was similarly induced on FGIN-1-27 treatment (Fig. 5a,b).

Increased amino acid synthesis and transport is characteristic of the amino acid starvation response (AAR)^20^. We, therefore, tested whether FGIN-1-27 indeed induced phosphorylation of eIF2α and translation of ATF4. FGIN-1-27 treatment led to a 4.5-fold increase in eIF2α phosphorylation and 2.8-fold increase in ATF4 translation compared to DMSO controls (Figs. 5c and S2a). As a result, we saw a significant increase in amino acids measured at 72 hours post compound treatment (Fig. 5d). Induction of ATF4 can be an indication of oxidative stress and we measured reactive oxygen species (ROS) using the cell permeant reagent 2′,7′ –dichlorofluorescin diacetate (DCFDA). We detected a dose dependent increase in intracellular ROS levels at 24-hour post treatment with FGIN-1-27 (Fig. 5e). Pre-treatment of T cells with an antioxidant, N-acetyl cysteine (NAC) only induced partial rescue (14% at 10 µM FGIN-1-27; Fig. 5f) of defective IL-17 production, demonstrating that other pathways besides oxidative stress may be regulating the effect of FGIN-1-27 on Th17 cell differentiation. FGIN-1-27 treatment showed a dose-dependent decrease in phosphorylation of S6 ribosomal protein (Ser 235/236) and mTOR (Ser 2448) (Fig. S2b,c). Thus, transcriptomic and metabolic profiling suggested that FGIN-1-27 treatment is regulating metabolic pathways such as glycolysis and amino acid synthesis and transport to regulate Th17 differentiation.

### Lipidome of Th17 cells is altered on treatment with FGIN-1-27

**Figure 6:**
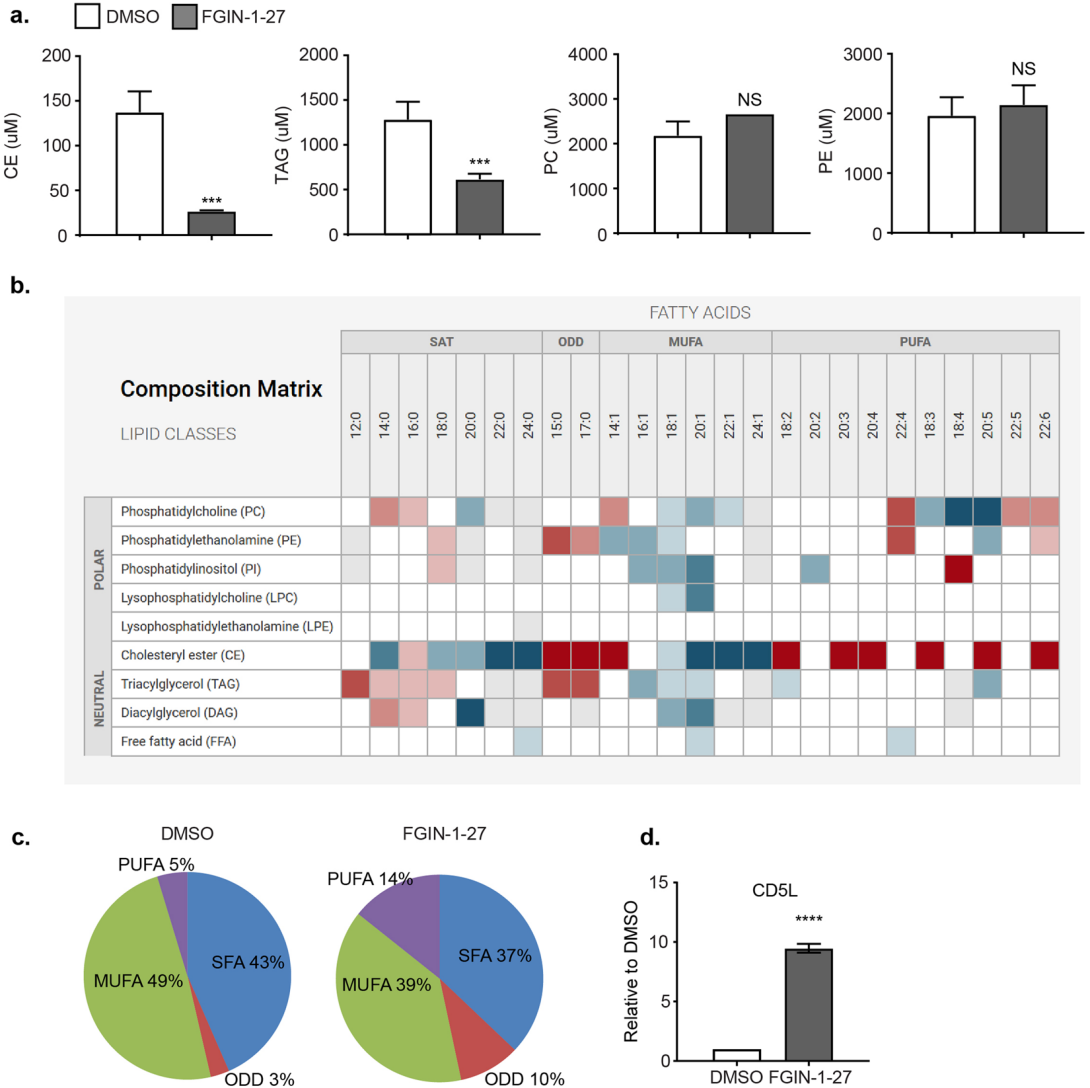
Lipidome is altered on FGIN-1-27 treatment. (**a**) Quantitation of cholesterol esters, triacylglycerols (TAGs), phosphatidylethanolamine (PE) and phosphatidylcholine (PC) in Th17 cells 72 hours after treatment with FGIN-1-27. The error bars represent SD. (**b**) Heat map showing fatty acid composition (mole percent) for different lipid classes after treatment with FGIN-1-27. (**c**) Pie charts showing proportions of different fatty acid classes namely PUFAs, MUFAs, ODD and SFAs within the cholesterol esters. (**d**) Transcript analysis for CD5L 72 hours post treatment with FGIN-1-27. The error bars represent SD.

Specific cholesterol precursors like desmosterol and oxysterols, such as 7β, 27-dihydroxycholesterol (7β, 27-OHC), have been identified as ligands for RORγt^21,22^ and shown to modulate Th17 differentiation. For this reason, and because TSPO has been implicated in cholesterol transport and metabolism in the mitochondria^23^, we analyzed free cholesterol and its precursors in T cells differentiated towards the Th17 lineage to determine whether FGIN-1-17 could act by modulating levels of cholesterol analogs know to modulate RORγt activity. We readily detected free cholesterol and its precursors such as 7-dehydrocholesterol and desmosterol in Th17 cells. We also detected low levels of other cholesterol precursors such as lanosterol, lathosterol, β-Sitosterol, campesterol and squalene. However, the levels of the measured cholesterol, its precursors and oxysterols, such as 7α hydoxycholesterol (7a-HC), were comparable in DMSO and FGIN-1-27 treated T cells (Table S1). Next, we extended our studies to lipid metabolites and profiled the lipidome of Th17 cells treated with DMSO or FGIN-1-27. We detected 1300 lipid metabolites from Th17 cells, of which 39.6% showed differential levels on FGIN-1-27 treatment (p < 0.05; Table S1). We readily detected 14 classes of lipids belonging to the three groups namely neutral lipids, phospholipids and sphingolipids, and found that cholesterol esters were dramatically down-regulated (5 folds) on FGIN-1-27 treatment (Fig. 6a,b). We also detected a 2-fold reduction in triacylglycerides (TAGs) on FGIN-1-27 treated T cells. Lipids such as phosphatidylethanolamine (PE) and phosphatidylcholine (PC) showed an upward trend and were modestly increased (not statistically significant) on FGIN-1-27 treatment (Fig. 6a,b).

Fatty acid composition (mole percent) analysis revealed that within cholesterol esters, saturated fatty acids (SFA) and mono-unsaturated fatty acids (MUFAs) were significantly down-regulated on FGIN-1-27 treatment whereas poly-unsaturated fatty acids (PUFAs) and odd-chain fatty acids (ODDs) were increased (Fig. 6b,c). Accordingly, we detected a substantial up-regulation of CD5L (CD5-like; Fig. 6d), a protein known to mediate Th17 pathogenesis by altering fatty acid composition and cholesterol biosynthesis^24^, on FGIN-1-27 treatment, further implicating that the fatty acid profile and Th17 pathogenicity is dramatically altered upon FGIN-1-27 treatment. As previously suggested for the CD5L^24^, the effects of FGIN-1-27 on lipid metabolism might modulate ligand availability for RORγt, which is a master regulator of Th17 differentiation.

## Discussion

To our knowledge, this is the first report demonstrating that a small molecule developed as a tracer for diagnostic imaging and shown to have anti-anxiety effects could be re-purposed to treat an autoimmune disease. The small molecule FGIN-1-27 has been reported to target TSPO^18^. We report that the effect of FGIN-1-27 on Th17 cell differentiation is independent of binding to its reported target, TSPO. There was no correlation between binding of FGIN-1-27 to TSPO in a biochemical assay with its cellular effects on IL-17 production. A recent study demonstrated that ligand residence time or the time a ligand interacts with its target namely TSPO is a more reliable measure that could be used to predict efficacy of TSPO ligands^25^. Even though we did not measure TSPO residence time for FGIN-1-27, we used genetic mouse models to rule out the role of TSPO in Th17 biology. Our data is in alignment with previous studies that have reported that biological effect of TSPO ligands on steroidogenesis can be independent of TSPO^19,23,26,27^. We explored if selective effects on TSPO2, a TSPO (TSPO1) paralog could perhaps explain the lack of FGIN-1-27 activity in the KO (TSPO1 KO) mouse cells. However, we did not detect expression of TSPO2 in Th17 cells confirming published reports that expression of TSPO2 is limited to erythrocytes^28^ (Data not shown).

**Figure 7:**
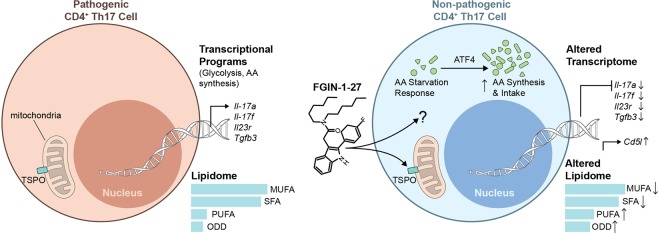
FGIN-1-27 is a potent repressor of Th17 differentiation program. (**a**) FGIN-1-27 ameliorates autoimmunity by metabolic reprogramming of pathogenic Th17 cells. Effect of FGIN-1-27 on Th17 cells is independent of TSPO, its reported ligand. FGIN-1-27 altered the Th17 differentiation program at multiple steps: (**a**) reducing glycolytic enzymes and glycolytic intermediates (**b**) increasing phosphorylation of eIF2α and translation of ATF4 resulting in activation of AAR (**c**) altering composition and balance of fatty acids.

While we do not have a complete understanding of the mechanism of action in Th17 cells, our data shows that FGIN-1-27 altered the Th17 differentiation program at multiple steps: (a) reducing glycolytic enzymes and glycolytic intermediates (b) increasing phosphorylation of eIF2α and translation of ATF4 resulting in activation of AAR (c) altering composition and balance of fatty acids and possibly, regulating ligand availability for RORγt (Fig. 7). Multiple reports have shown that T cells on activation through the TCR or antigen reprogram their metabolism to meet the increased energy demand for proliferation and effector function^14,29,30^. Different T cell subsets use distinct metabolic programs to fuel increase energy needs for T cell proliferation and effector function. Th17 cells are heavily dependent on glycolysis for their development and maintenance^13,14,30^. Likewise, anergic T cells and dysfunctional tumor infiltrating lymphocytes are reported to have defects in glycolytic pathway^31–33^ and deficiency in glucose transporter glut1 has been linked with decreased effector function and protection from inflammatory diseases^34^. We detected reduction in expression of glucose transporter *Glut3* (GEO accession GSE128308) in addition to all the transcripts encoding enzymes glycolytic enzymes leading us to conclude that FGIN-1-27 treatment alters the glycolytic pathway in Th17 cells, thus demonstrating that a small molecule can be used to alter the glycolytic machinery in a cell to achieve a desirable immune response.

Our data are in accordance with a recently published report where ATF4 promotes amino acid synthesis and deficiency in ATF4 resulted in increased Th17 responses^35^. Previously, activation of the AAR by the small molecule halofuginone has been shown to down-regulate Th17 responses^20^. T cell activation leads to increase in protein synthesis and up-regulation of multiple amino acid transporters to enhance uptake of amino acids^29^. We detected increased phosphorylation of eIF2α and translation of ATF4 on FGIN-1-27 treatment. Induction of ATF4 enhanced de novo amino acid synthesis by upregulating amino acid transporters such as Slc1a4, Slc7a3 and Asns. In addition to promoting anabolic programs, ATF4 also promoted anaplerosis and cataplerosis by upregulating Gpt2 and Pck2.

In our study, FGIN-1-27 treatment limited the pathogenicity of Th17 cells without affecting protein or transcript levels for RORγt. We saw a downregulation in the expression of RORγt target genes (*Il17a*, *Il17f*, *Il23r*, *Lt4r1* and *Ccr6*) by FGIN-1-27. Lipid metabolism, specially cholesterol esters and triacylglycerols showed dramatic reduction on FGIN-1-27 treatment. We demonstrated that fatty acid compositions, PUFA/SFA balance and expression of CD5L were altered in FGIN-1-27 treated Th17 cells. Previously, PUFAs have been reported to decrease obesity associated Th17 mediated inflammation during colitis^36^. Similar loss of CD5L and regulation of lipid biosynthesis has been associated with acquisition of pathogenicity in Th17 cells^24^. Trafficking of encephalitogenic T cells at inflammatory loci is a critical step for disease development and progression. The fact that FGIN-1-27 delayed trafficking and invasion of FGIN-1-27-treated T cells to CNS leading to delayed onset and reduced severity of EAE suggest this compound could be further developed as a potential novel therapeutic avenue for treating Th17-mediated immunopathology for autoimmune diseases.

## Methods

### Mice

C57B/6 mice and 2D2 TCR transgenic mice were purchased from Jackson Lab for the studies. Generation of TSPO knockout mice has been previously described^19^. Animals were used between 7-12 weeks of age. All mice were maintained in accordance with the National Institutes of Health Guide for the Care and Use of Laboratory Animals and all animal experiments were approved by the NIH Division of Veterinary Resources and NCATS (National Center for Advancing Translational Sciences) and NCI (National Cancer Institute) Animal Care and Use Committee.

### Cell isolation and flow cytometry

Single cell suspensions were prepared from spleens and lymph nodes of mice by mechanical disruption. Red blood cells were lysed by ammonium chloride followed by CD4^+^ T cell purification using either CD4 microbeads (Miltenyi Biotec) or sorting for naïve CD4^+^ T cells (FACS Aria). 250,000 cells/well in a 48 well plate were polarized into Th17 cells using Th17 differentiation cocktail: IFNγ, IL-4, IL-12 neutralizing antibodies (10 μg/ml) and 10 ng/ml each of IL-6, IL-1b, IL-21 and TGF-β (0.5 ng/ml). Cells were treated with FGIN-1-27 (Tocris Bioscience) or DMSO at 18 hour and 48 hours post cell plating. On day 4 of culture, cells were re-stimulated with PMA-ionomycin in the presence of brefeldin A. Cells were subsequently stained with viability dye and surface stained for CD44, CD4 followed by permeabilization using cytofix/cytoperm (BD biosciences) and stained for intracellular IL-17. RORγt intranuclear staining was performed after fixation and permeabilization with a Foxp3/Transcription Factor Staining kit (eBioscience). Cells were acquired on BD Fortessa followed by analysis using Flowjo (Tree star Inc).

### RNA purification and quantitative PCR

Total RNA extractions were performed with the RNAeasy kit (Qiagen) followed by cDNA synthesis using High capacity cDNA Reverse Transcription kit (Applied Biosystems). Quantitative PCR was performed on a ViiA 7 (Applied Biosystems) using SYBR Fast qPCR master mix (Kapa Biosystems). The following qPCR probes were used. IL-17a (F): TCAGCGTGTCCAAACACTGAG, IL-17a (R): CGCCAAGGGAGTTAAAGACTT; Il-17f (F): TGCTACTGTTGATGTTGGGAC, IL-17f (R):CAGAAATGCCCTGGTTTTGGT; Lt4r1 (F): GATGCAGAAACGCACGGTC, Lt4r1 (R):ACTGCCAGTGATCGGTCCA; Rorc (F) TCCACTACGGGGTTATCACCT, Rorc (R) AGTAGGCCACATTACACTGCT; Ccr6 (F): TGGGCCATGCTCCCTAGAA, Ccr6 (R): GGTGAGGACAAAGAGTATGTCTG; CD5L (F): GGGGTTGACTGCAACGGAA, CD5L (R): GGCCATCTACTAGACGCACA, Il-23r (F): AACAACAGCTCGGATTTGGTAT IL-23r (R) ATGACCAGGACATTCAGCAGT

### Phospho ZAP70 and phospho Stat3 staining

Naïve CD4^+^ T cells were isolated from spleen and lymph nodes of C57BL/6 mice using naïve CD4 negative selection kit (StemCell) and activated under Th17-polarizing conditions in the presence of irradiated wild-type splenocytes at a 5:1 ratio, with anti-CD3 (2.5 μg/ml) (145-2C11, BioXCell), anti-IL-4 (10 μg/ml) (11B11, BioXCell), anti-IFNγ (10 μg/ml) (XMG1.2, BioXCell), mIL-6 (30 ng/ml) (Miltenyi Biotec) and hTGF-β1 (3 ng/ml) (StemCell). The following day, cells were treated with either DMSO or of FGIN-1-27 (10 μM). For phospho-ZAP70 analyses, cells were stained after 24 hours of treatment using 10 μg/ml of biotinylated anti-CD3 (145-2C11, BD Biosciences) and anti-CD28 (37.51, BD Biosciences) antibodies on ice for 15 minutes, washed, and stimulated in presence of 20 μg/ml of Streptavidin for 10 minutes at 37 degrees. Cells were then fixed in 2% PFA, permeabilized in Methanol/Acetone and stained with anti-phospho ZAP70 antibody (n3kobu5, eBioscience) and anti-CD4 (RM4–5, eBioscience). For phospho-STAT3 analyses, cells were cultured for additional 24 hours in IL-6 free media, before stimulation with 100 ng/ml of mIL-6 for 30 minutes. Cells were then fixed in 2% PFA, permeabilized in Methanol/Acetone and stained with anti-phospho-STAT3 antibody (4/P-STAT3, BD Biosciences) and anti-CD4. Dead cells were excluded using Live/Dead Amcyan (Thermo Fisher) staining prior to stimulation.

### Phospho S6 and phospho mTOR staining

For phospho-S6 analyses, cells were cultured for 3 hours in Th17 polarization media on anti-CD3/CD28 coated plates. Cells were then fixed after fixation and permeabilization with a Transcription Factor Staining kit (eBioscience) and stained with anti-phospho-S6 antibody (S235/236, Cell Signaling) and anti-CD4. Dead cells were excluded using Live/Dead stain prior to fixation. For mTOR analyses, anti-phospho-mTOR antibody was used (Ser2448, eBioscience).

### RNA-Seq

Total RNA extractions for RNA-seq were performed using the mirVana kit (Applied Biosystems). RNA concentration was measured using the Qubit RNA broad range assay in the Qubit Flurometer (Invitrogen) and RNA integrity was determined with Eukaryote Total RNA Nano Series II ChIP on a 2100 Bioanalyzer (Agilent). RNA-seq libraries were prepared using TruSeq RNA sample preparation kit (Illumina). In brief, oligo-dT purified mRNA was fragmented and subjected to first and second strand cDNA synthesis. cDNA fragments were blunt ended, ligated to Illumina adaptors and PCR amplified to enrich for the fragments ligated to adaptors. The resulting cDNA libraries were verified and quantified on Agilent Bioanalyzer and single-end 96 cycle sequencing was conducted (Illumina).

### RNA seq data analysis

Reads were aligned to the mm10 genome and the GRCm38 v87 transcriptome with STAR v2.5.2a using default parameters and output tracks normalized by RPM (–outWigNorm RPM). RSEM v1.3 was used to calculate gene counts and TPMs on the transcriptome-aligned reads as previously described^37^. Differential expression analysis was performed in R v3.5.2, using DESeq. 2 v1.22.2. Pathway enrichment analysis was performed on the 587 differentially expressed genes using InnateDB v5.4, against the INOH, KEGG and REACTOME databases^38^. Heatmaps were generated with the R package, heatmap. Integrative Genomics Viewer v2.4.19 was used for visualization of normalized bigwig tracks. The generation of coverage tracks from the T cell RNA-seq data of Ciofani *et al*.^15^, (GEO accession GSE40918), was performed with STAR as described above, except as unstranded (–outWigStrand Unstranded).

### 2D2 Th17 cell differentiation, passive induction of EAE and disease scoring

Naïve CD4^+^ T cells (CD4^+^V_β_11^+^CD62L^hi^) were sorted from the spleens and lymph nodes of 2D2 TCR transgenic mice and polarized towards Th17 lineage in the presence of irradiated splenocytes (5:1 ratio) and 2.5 μg/ml anti-CD3 (145-2C11, BioXCell), 20 μg/ml anti-IL-4 (11B11, BioXCell), 20 μg/ml anti-IFNγ (XMG1.2, BioXCell), 30 ng/ml mIL-6 and 3 ng/ml hTGF-β1 (Miltenyi Biotec). The following day, cells were treated with either DMSO or FGIN-1-27 (10 μM) and IL-23 (10 ng/ml; R&D Systems) was added after 60 h of activation. On day 5 of culture, cells were reactivated on plates precoated with 2 μg/ml of anti-CD3 and anti-CD28 (PV1, BioXCell) for 48 h, and 5 million cells were adoptively transferred to recipient mice to induce passive-transfer EAE. Classical EAE symptoms were scored daily according to standard criteria: 0, asymptomatic; 1, flaccid tail; 2, hind-limb weakness and impaired righting ability; 3, hind-limb paralysis; 4, front- and hind-limb paralysis; 5, moribund or death.

### Active induction of EAE

Active EAE was induced by immunization with the myelin oligodendrocyte glycoprotein (MOG) peptide 35–55 (MEVGWYRSPFSRVVHLYRNGK). 100 μg MOG_35–55_ peptide was emulsified in complete Freund’s adjuvant supplemented with *M. tuberculosis* extract H37Ra (Difco) and injected subcutaneously. Mice received 150 ng pertussis toxin (List Biological Laboratories) intraperitoneally on days 0 and 2. Pharmaceutical-grade FGIN-1-27 was obtained from TOCRIS (Catalog Number: 142720-24-9). A 3 mg/mL solution of FGIN-1-27 was prepared in a vehicle of 10% ethanol, 50% of 20% beta HP-CD (Sigma-Aldrich, Catalog # H107) in water, and 40% PEG 300 (Sigma-Aldrich, Catalog # 202371). Mice were injected twice daily (8 hours apart) via intraperitoneal route with 200 µl of vehicle or 30 mg/kg of FGIN-1-27 for 7 days. Twice-daily injections (IP) were alternated between the left and right quadrant of the lower abdomen. Classical EAE symptoms were scored daily according to standard criteria: 0, asymptomatic; 1, flaccid tail; 2, hind limb weakness and impaired righting ability; 3, hind limb paralysis; 4, front and hind limb paralysis; 5, moribund or death.

### Western blot

Cell lysates were prepared by lysis in RIPA buffer containing protease and phosphatase inhibitors and immunoblotting was performed by standard methods. The following antibodies were used: TSPO (ab109497, Abcam), ATF4(#11815, Cell signaling) and phospho eiF2a (#3398, Cell Signaling).

### TSPO binding studies

FGIN-1-27 were tested at 8 doses (0.3–40 µM) in the TSPO (BZDp) antagonist radioligand displacement assay at Eurofins Cerep (France). Radiolabeled PK11195 was used as a control and TSPO binding was calculated as a percent inhibition of control specific binding using the formula 100-(measured specific binding*100/control specific binding).

### Detection of intracellular metabolites

8 million cells treated with either DMSO or FGIN-1-27 were used for extraction of intracellular metabolites. The cells were washed twice with 5% mannitol and treated with methanol to inactivate the enzymes. Next, the cell extract was treated with Milli-Q water containing internal standards (H3304-1002, Human Metabolome Technologies, inc., Tsuruoka, Japan) and left at rest for another 30 s. The extract was obtained and centrifuged at 2,300 ×*g* and 4 °C for 5 min and then 800 µL of upper aqueous layer was centrifugally filtered through a Millipore 5-kDa cutoff filter at 9,100 ×*g* and 4 °C for 120 min to remove proteins. The filtrate was centrifugally concentrated and re-suspended in 50 µL of Milli-Q water for CE-MS analysis. Cationic compounds were measured in the positive mode of CE-TOFMS and anionic compounds were measured in the positive and negative modes of CE-MS/MS according to the methods developed by Soga, *et al*.^39,40^. Peaks detected by CE-TOFMS and CE-MS/MS were extracted using automatic integration software (MasterHands, Keio University, Tsuruoka, Japan and MassHunter Quantitative Analysis B.04.00, Agilent Technologies, Santa Clara, CA, USA, respectively) in order to obtain peak information including *m/z*, migration time (MT), and peak area. The peaks were annotated with putative metabolites from the HMT metabolite database based on their MTs in CE and *m*/*z* values determined by TOFMS. The tolerance range for the peak annotation was configured at ±0.5 min for MT and ±10 ppm for *m/z*. In addition, concentrations of metabolites were calculated by normalizing the peak area of each metabolite with respect to the area of the internal standard and by using standard curves, which were obtained by three-point calibrations. Hierarchical cluster analysis (HCA) and principal component analysis (PCA) were performed by Human Metabolome proprietary software, PeakStat and SampleStat, respectively. Detected metabolites were plotted on metabolic pathway maps using VANTED (Visualization and Analysis of Networks containing Experimental Data) software.

### Lipidomics

Th17 cells were differentiated from naïve purified CD4 T cells and treated with either FGIN-1-27 (10 µM) or DMSO during polarization. 10 million cells/treatment were harvested and snap frozen. Lipids were extracted from samples using dichloromethane and methanol in a modified Bligh-Dyer extraction in the presence of internal standards with the lower, organic, phase being used for analysis. The extracts were concentrated under nitrogen and reconstituted in 0.25 mL of dichloromethane:methanol (50:50) containing 10 mM ammonium acetate. The extracts were placed in vials for infusion-MS analyses, performed on a SelexION equipped Sciex 5500 QTRAP using both positive and negative mode electrospray. Each sample was subjected to 2 analyses, with IMS-MS conditions optimized for lipid classes monitored in each analysis. The 5500 QTRAP was operated in MRM mode to monitor the transitions for over 1,100 lipids from up to 14 lipid classes. Individual lipid species were quantified based on the ratio of signal intensity for target compounds to the signal intensity for an assigned internal standard of known concentration. Lipid class concentrations were calculated from the sum of all molecular species within a class, and fatty acid compositions were determined by calculating the proportion of individual fatty acids within each class.

### Statistics

Data were analyzed using a 2-tailed Student’s t-test and p < 0.05 was considered statistically significant.

## Supplementary information

supplementary information.

supplementary dataset.

## References

[1] Harrington LE (2005). Interleukin 17-producing CD4+ effector T cells develop via a lineage distinct from the T helper type 1 and 2 lineages. Nat. Immunol..

[2] Mangan PR (2006). Transforming growth factor-beta induces development of the T(H)17 lineage. Nat..

[3] Cua DJ (2003). Interleukin-23 rather than interleukin-12 is the critical cytokine for autoimmune inflammation of the brain. Nat..

[4] Gaffen SL, Hernandez-Santos N, Peterson AC (2011). IL-17 signaling in host defense against Candida albicans. Immunol. Res..

[5] Romani L (2011). Immunity to fungal infections. Nat. Rev. Immunol..

[6] Bettelli E (2006). Reciprocal developmental pathways for the generation of pathogenic effector TH17 and regulatory T cells. Nat..

[7] Lee Y (2012). Induction and molecular signature of pathogenic TH17 cells. Nat. Immunol..

[8] McGeachy MJ (2009). The interleukin 23 receptor is essential for the terminal differentiation of interleukin 17-producing effector T helper cells *in vivo*. Nat. Immunol..

[9] Curtsinger JM (1999). Inflammatory cytokines provide a third signal for activation of naive CD4+ and CD8+ T cells. J. Immunol..

[10] Yamane H, Paul WE (2013). Early signaling events that underlie fate decisions of naive CD4(+) T cells toward distinct T-helper cell subsets. Immunol. Rev..

[11] Fox CJ, Hammerman PS, Thompson CB (2005). Fuel feeds function: energy metabolism and the T-cell response. Nat. Rev. Immunol..

[12] van der Windt GJ, Pearce EL (2012). Metabolic switching and fuel choice during T-cell differentiation and memory development. Immunol. Rev..

[13] Shi LZ (2011). HIF1alpha-dependent glycolytic pathway orchestrates a metabolic checkpoint for the differentiation of TH17 and Treg cells. J. Exp. Med..

[14] Gerriets VA (2015). Metabolic programming and PDHK1 control CD4+ T cell subsets and inflammation. J. Clin. Invest..

[15] Ciofani M (2012). A validated regulatory network for Th17 cell specification. Cell.

[16] Rupprecht R (2010). Translocator protein (18 kDa) (TSPO) as a therapeutic target for neurological and psychiatric disorders. Nat. Rev. Drug. Discov..

[17] Romeo E (1992). 2-Aryl-3-indoleacetamides (FGIN-1): a new class of potent and specific ligands for the mitochondrial DBI receptor (MDR). J. Pharmacol. Exp. Ther..

[18] Lima-Maximino MG, Cueto-Escobedo J, Rodriguez-Landa JF, Maximino C (2018). FGIN-1-27, an agonist at translocator protein 18kDa (TSPO), produces anti-anxiety and anti-panic effects in non-mammalian models. Pharmacol. Biochem. Behav..

[19] Tu LN (2014). Peripheral benzodiazepine receptor/translocator protein global knock-out mice are viable with no effects on steroid hormone biosynthesis. J. Biol. Chem..

[20] Sundrud MS (2009). Halofuginone inhibits TH17 cell differentiation by activating the amino acid starvation response. Sci..

[21] Soroosh P (2014). Oxysterols are agonist ligands of RORgammat and drive Th17 cell differentiation. Proc. Natl Acad. Sci. USA.

[22] Hu X (2015). Sterol metabolism controls T(H)17 differentiation by generating endogenous RORgamma agonists. Nat. Chem. Biol..

[23] Selvaraj V, Stocco DM (2015). The changing landscape in translocator protein (TSPO) function. Trends Endocrinol. Metab..

[24] Wang C (2015). CD5L/AIM Regulates Lipid Biosynthesis and Restrains Th17 Cell Pathogenicity. Cell.

[25] Costa B (2016). TSPO ligand residence time: a new parameter to predict compound neurosteroidogenic efficacy. Sci. Rep..

[26] Tu LN, Zhao AH, Stocco DM, Selvaraj V (2015). PK11195 effect on steroidogenesis is not mediated through the translocator protein (TSPO). Endocrinol..

[27] Selvaraj V, Stocco DM, Tu LN (2015). Minireview: translocator protein (TSPO) and steroidogenesis: a reappraisal. Mol. Endocrinol..

[28] Fan J, Rone MB, Papadopoulos V (2009). Translocator protein 2 is involved in cholesterol redistribution during erythropoiesis. J. Biol. Chem..

[29] Sinclair LV (2013). Control of amino-acid transport by antigen receptors coordinates the metabolic reprogramming essential for T cell differentiation. Nat. Immunol..

[30] Buck MD, O’Sullivan D, Pearce EL (2015). T cell metabolism drives immunity. J. Exp. Med..

[31] Gemta Lelisa F., Siska Peter J., Nelson Marin E., Gao Xia, Liu Xiaojing, Locasale Jason W., Yagita Hideo, Slingluff Craig L., Hoehn Kyle L., Rathmell Jeffrey C., Bullock Timothy N. J. (2019). Impaired enolase 1 glycolytic activity restrains effector functions of tumor-infiltrating CD8+T cells. Science Immunology.

[32] Chang CH (2015). Metabolic Competition in the Tumor Microenvironment Is a Driver of Cancer Progression. Cell.

[33] Bengsch B (2016). Bioenergetic Insufficiencies Due to Metabolic Alterations Regulated by the Inhibitory Receptor PD-1 Are an Early Driver of CD8(+) T Cell Exhaustion. Immun..

[34] Macintyre AN (2014). The glucose transporter Glut1 is selectively essential for CD4 T cell activation and effector function. Cell Metab..

[35] Yang X (2018). ATF4 Regulates CD4(+) T Cell Immune Responses through Metabolic Reprogramming. Cell Rep..

[36] Monk JM (2012). Dietary n-3 polyunsaturated fatty acids (PUFA) decrease obesity-associated Th17 cell-mediated inflammation during colitis. PLoS One.

[37] Escobar TM (2014). miR-155 activates cytokine gene expression in Th17 cells by regulating the DNA-binding protein Jarid2 to relieve polycomb-mediated repression. Immun..

[38] Breuer K (2013). InnateDB: systems biology of innate immunity and beyond–recent updates and continuing curation. Nucleic Acids Res..

[39] Soga T (2003). Quantitative metabolome analysis using capillary electrophoresis mass spectrometry. J. Proteome Res..

[40] Soga T, Heiger DN (2000). Amino acid analysis by capillary electrophoresis electrospray ionization mass spectrometry. Anal. Chem..

