# Supplementary material for: Anxiolytic Drug FGIN-1-27 Ameliorates Autoimmunity by Metabolic Reprogramming of Pathogenic Th17 Cells
Source: Sci Rep. 2020 Feb 28;10:3766. doi: 10.1038/s41598-020-60610-5 (PMC7048748; doi:10.1038/s41598-020-60610-5)
Supplement: Supplementary file 1 — supplementary information. [file 41598_2020_60610_MOESM1_ESM.pdf]

# Anxiolytic Drug FGIN-1-27 Ameliorates Autoimmunity by Metabolic Reprogramming of Pathogenic Th17 Cells

Anju Singh[1], Myagmarjav Dashnyam[1], Bryan Chim[2], Thelma M. Escobar[2], Andrés E. Dulcey[1], Xin Hu[1], Kelli M. Wilson[1], Prasanthi P Koganti[3], Camille A. Spinner[4], Xin Xu[1], Ajit Jadhav[1], Noel Southall[1], Juan Marugan[1], Vimal Selvaraj[3], Vanja Lazarevic*[4], Stefan A. Muljo*[2], and Marc Ferrer*[1]

Figure S1

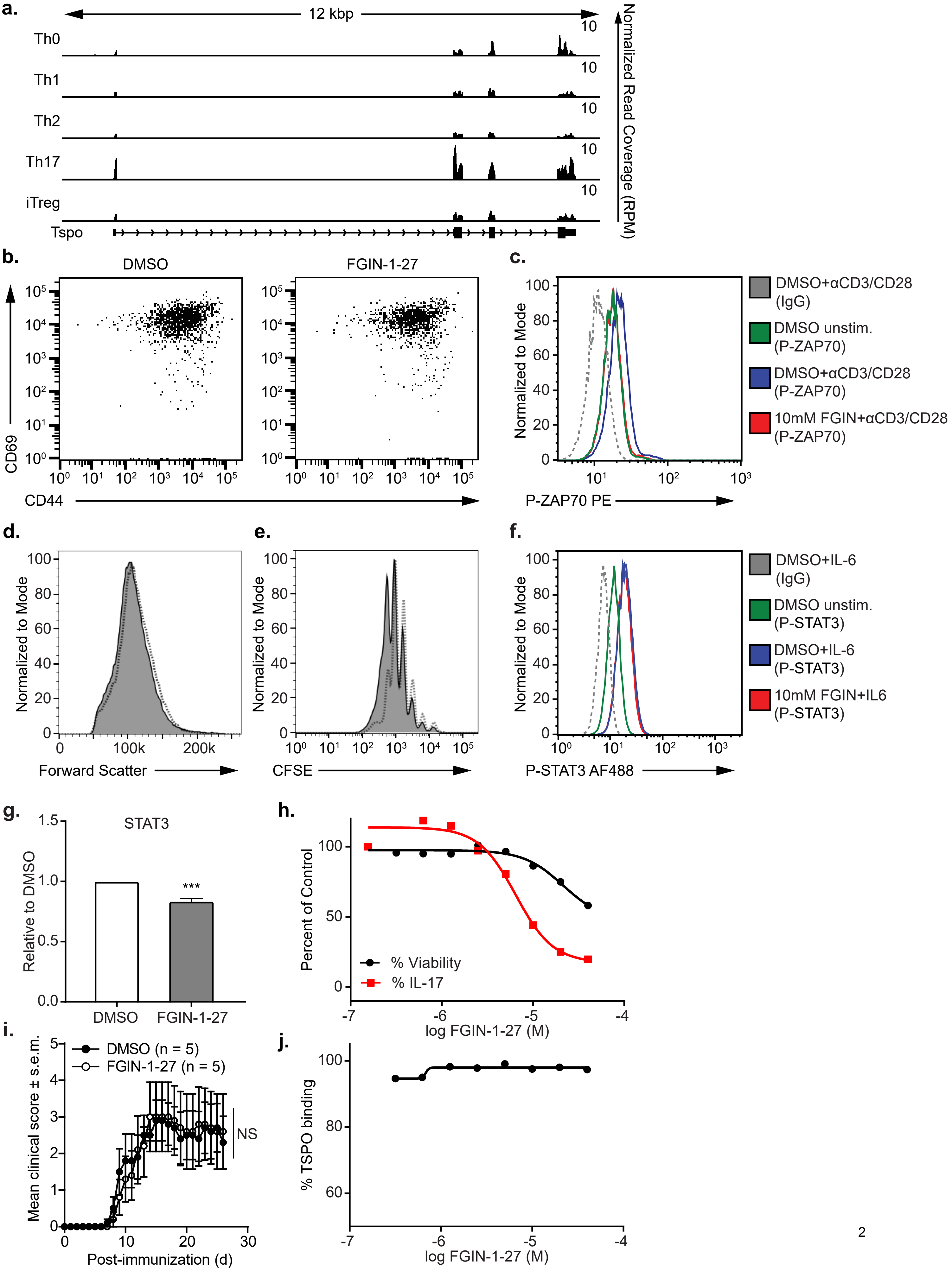

a.
12 kbp
Th0
Th1
Th2
Th17
iTreg
Tspo
10
Normalized Read Coverage (RPM)
b.
DMSO
FGIN-1-27
CD69
CD44
c.
Normalized to Mode
P-ZAP70 PE
DMSO+αCD3/CD28 (IgG)
DMSO unstim. (P-ZAP70)
DMSO+αCD3/CD28 (P-ZAP70)
10mM FGIN+αCD3/CD28 (P-ZAP70)
d.
Forward Scatter
e.
CFSE
f.
P-STAT3 AF488
DMSO+IL-6 (IgG)
DMSO unstim. (P-STAT3)
DMSO+IL-6 (P-STAT3)
10mM FGIN+IL6 (P-STAT3)
g.
STAT3
Relative to DMSO
***
DMSO
FGIN-1-27
h.
Percent of Control
% Viability
% IL-17
log FGIN-1-27 (M)
i.
Mean clinical score ± s.e.m.
DMSO (n = 5)
FGIN-1-27 (n = 5)
NS
Post-immunization (d)
j.
% TSPO binding
log FGIN-1-27 (M)

## Supplementary Figures

### Fig S1

(a) Genomic browser screenshot of Tspo depicting normalized RNA-seq read coverage (RPM) in different T cell subsets. Data were downloaded from NCBI GEO (accession number GSE40918) and processed as in Figure 4A.

(b) Dot plots showing DMSO or FGIN-1-27 treated cells stained for surface CD4, CD44 and CD69 after 24 hours of treatment.

(c) Intracellular phospho-ZAP70 or isotype control staining of Th17 cells treated with DMSO or FGIN-1-27 for 24 hours and re-stimulated in presence or absence of anti-CD3 and anti-CD28 antibodies for 10 minutes.

(d) Histograms showing cell size (forward scatter) for DMSO (filled grey) or FGIN-1-27 treated (dotted) $CD4^+$ T cells at 24 hours post treatment. The plot is gated on live cells

(e) Histograms showing CFSE dilution for DMSO (filled grey) or FGIN-1-27 treated (dotted) CD4 T cells at 72 hours post treatment. The plot is gated on live $CD44^{high}$ CD4 $T^+$ cells.

(f) Intracellular phospho-STAT3 or isotype control staining of Th17 cells treated with DMSO or FGIN-1-27 for 24 hours and stimulated in presence or absence of mIL-6 for 30 minutes.

(g) Transcript levels for *Stat3* after 24 hours of treatment with DMSO or FGIN-1-27 quantified by qPCR. Data was normalized to a housekeeping gene, β-actin.

(h) Dose response plot showing effect of FGIN-1-27 on intracellular IL-17 and viability in pre-differentiated Th17 cells after re-stimulation. Data is normalized against DMSO (100%)

(i) Mean clinical scores of mice following active induction of EAE. Mice were injected i/p twice daily with vehicle or FGIN-1-27 for 7 days. Data are pooled from 5 mice/group. The error bars represent SEM.

(j) Binding of FGIN-1-27 to TSPO in a radioligand displacement assay. Inhibition of binding of control (radiolabeled PK-11195) was used to determine binding of FGIN-1-27 to TSPO

## Figure S2

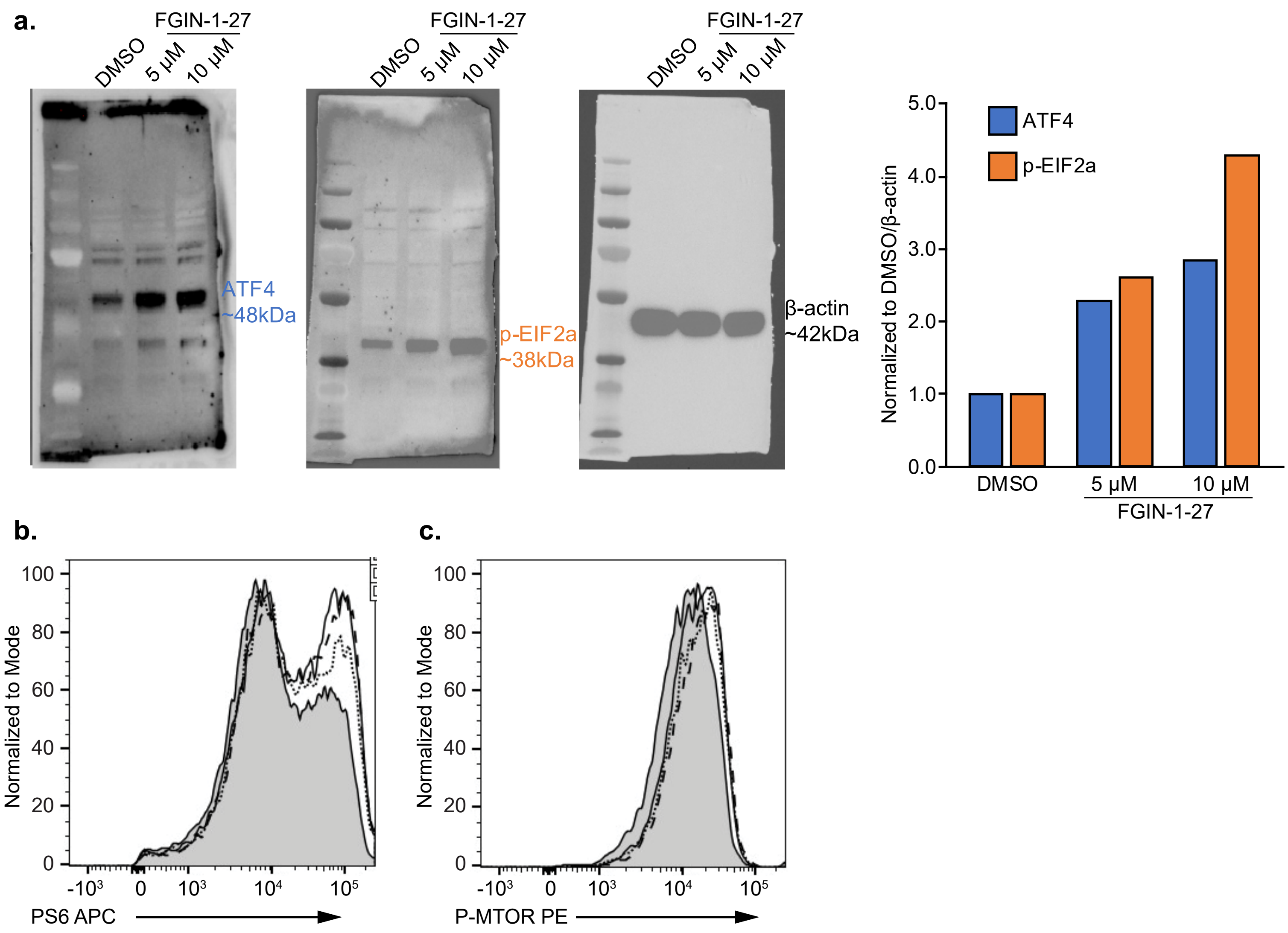
a.
FGIN-1-27
DMSO
5 μM
10 μM
ATF4
~48kDa
p-EIF2a
~38kDa
β-actin
~42kDa
ATF4
p-EIF2a
Normalized to DMSO/β-actin
5.0
4.0
3.0
2.0
1.0
0.0
DMSO
5 μM
10 μM
FGIN-1-27
b.
c.
Normalized to Mode
100
80
60
40
20
0
-10³
0
10³
10⁴
10⁵
PS6 APC
P-MTOR PE

## Fig S2

(a) Western blot analysis for phosphorylated eIF2α and ATF4 24 hours after treatment with DMSO or FGIN-1-27 (5 and 10 μM).

(b) Intracellular phospho S6 (Ser 235/236) Th17 cells treated with DMSO or FGIN-1-27 (10, 5 and 2.5 μM) for 3 hours. The black line represents DMSO and the grey filled histogram represent FGIN-1-27 (10μM). The dotted lines (5μM) and the dashed line represents staining after 2.5μM of FGIN-1-27 treatment.

(c) Intracellular phospho mTOR (Ser 2448) Th17 cells treated with DMSO or FGIN-1-27 (10, 5 and 2.5 μM) for 24 hours. The black line represents DMSO and the grey filled histogram represent FGIN-1-27 (10μM). The dotted lines (5μM) and the dashed line represents staining after 2.5μM of FGIN-1-27 treatment.

## Table S1

(a) Indicated sterols were measured in Th17 cells treated with DMSO or FGIN-1-27 for 72 hours

(b) Table showing 1300 lipid metabolites detected from Th17 cells, of which 39.6% showed differential levels on FGIN-1-27 treatment
